# Secretome of Peripheral Blood Mononuclear Cells Enhances Wound Healing

**DOI:** 10.1371/journal.pone.0060103

**Published:** 2013-03-22

**Authors:** Michael Mildner, Stefan Hacker, Thomas Haider, Maria Gschwandtner, Gregor Werba, Caterina Barresi, Matthias Zimmermann, Bahar Golabi, Erwin Tschachler, Hendrik Jan Ankersmit

**Affiliations:** 1 Department of Dermatology, Medical University Vienna, Vienna, Austria; 2 Department of Plastic Surgery, Medical University Vienna, Vienna, Austria; 3 Christian Doppler Laboratory for Cardiac and Thoracic Diagnosis and Regeneration, Vienna, Austria; 4 Department of Thoracic Surgery, Medical University Vienna, Vienna, Austria; 5 Department of Surgery, Medical University Vienna, Vienna, Austria; 6 Centre de Recherches et dlnvestigations Epidermiques et Sensorielles (CE.R.I.E.S.), Neuilly, France; University Hospital Hamburg-Eppendorf, Germany

## Abstract

Non-healing skin ulcers are often resistant to most common therapies. Treatment with growth factors has been demonstrated to improve closure of chronic wounds. Here we investigate whether lyophilized culture supernatant of freshly isolated peripheral blood mononuclear cells (PBMC) is able to enhance wound healing. PBMC from healthy human individuals were prepared and cultured for 24 hours. Supernatants were collected, dialyzed and lyophilized (SEC^PBMC^). Six mm punch biopsy wounds were set on the backs of C57BL/6J-mice and SEC^PBMC^ containing emulsion or controls were applied daily for three days. Morphology and neo-angiogenesis were analyzed by H&E-staining and CD31 immuno-staining, respectively. *In vitro* effects on diverse skin cells were investigated by migration assays, cell cycle analysis, and tube formation assay. Signaling pathways were analyzed by Western blot analysis. Application of SEC^PBMC^ on 6 mm punch biopsy wounds significantly enhanced wound closure. H&E staining of the wounds after 6 days revealed that wound healing was more advanced after application of SEC^PBMC^ containing emulsion. Furthermore, there was a massive increase in CD31 positive cells, indicating enhanced neo-angiogenesis. In primary human fibroblasts (FB) and keratinocytes (KC) migration but not proliferation was induced. In endothelial cells (EC) SEC^PBMC^ induced proliferation and tube-formation in a matrigel-assay. In addition, SEC^PBMC^ treatment of skin cells led to the induction of multiple signaling pathways involved in cell migration, proliferation and survival. In summary, we could show that emulsions containing the secretome of PBMC derived from healthy individuals accelerates wound healing in a mouse model and induce wound healing associated mechanisms in human primary skin cells. The formulation and use of such emulsions might therefore represent a possible novel option for the treatment of non-healing skin ulcers.

## Introduction

The skin is the largest organ of the human body. It covers and protects the underlying organs from ultraviolet radiation, mechanical and chemical damage, invading microorganisms and excessive water loss [1]. Due to this essential function, skin wounds need to be efficiently repaired within a very short time frame. Optimum healing of cutaneous wounds requires a well-orchestrated integration of complex biological and molecular events, including cell migration and proliferation, extracellular matrix deposition and remodeling as well as neo-angiogenesis [2]–[4]. Unfortunately, adequate healing of skin wounds, in particular in the elderly population or in diabetic patients, is often impaired, leading to increased morbidity [5]. The seminal work by Holzinger *et al.* has shown that autologous transplantation of mononuclear cells effectively initiates and improves granulation and epithelialization of skin ulcers [6]. In addition, topic application of a mixture of peripheral blood mononuclear cells (PBMC) together with basic fibroblast growth factor also resulted in a dramatic improvement in the treatment of a diabetic gangrene [7]. Several reports showed that transplantation of stem cells accelerates wound re-epithelialization and neovascularization in various models [8]–[10]. Recently, it has been demonstrated that the efficacy of a stem cell based therapy is dependent on soluble factors produced by these cells, since the secretome of stem cell cultures is sufficient to accelerate cutaneous wound healing [11]–[13].

The idea of using conditioned medium as a therapeutic agent evolved in the field of stem cell research and originated from cell based therapy of myocardial infarction. Many of the regenerative effects seen after administration of stem cells were shown to be rather mediated via a paracrine signaling than by direct cellular interactions [14], [15]. Conditioned culture medium, containing the secretome of mesenchymal stem cells, is rich in angiogenic and chemotactic factors [16], [17]. In addition, first evidence emerges that stem cell conditioned medium has immunosuppressive properties [18], [19]. We have previously shown that infusion of cultured irradiated apoptotic PBMC suspensions in a rat acute myocardial infarction (AMI) model restored long-term cardiac function [20], [21]. In a further study, we demonstrated that the regenerative function of these cells was solely mediated via soluble factors produced by the cells [22]. These highly active culture supernatants not only showed positive effects in a rodent and porcine AMI model, but also displayed cytoprotective functions on human primary cardiomyocytes [22].

Based on these beneficial pathophysiological concepts we speculated whether an emulsion containing lyophilized culture supernatants of freshly isolated unstimulated PBMC (SEC^PBMC^) is able to enhance skin wound healing in a mouse model *in vivo* and whether it is able to activate wound healing associated mechanisms in primary human skin cells *in vitro*.

## Methods

### Ethics statement

Animal experiments were approved by the committee for animal research, Medical University of Vienna (vote: 66.009/0108-II/10b/2009).

Human peripheral blood mononuclear cells (PBMC) were obtained from young healthy volunteers. The study was approved by the local ethics committee (Medical University of Vienna, vote: EK-Nr 2010/034), and informed written consent was obtained from all volunteers.

### Mice

C57BL/6J mice were purchased (Taconic, Ry, Denmark) and kept at the Division of Biomedical Research at the Medical University of Vienna. Three months old mice were used for the wounding experiments.

### Wounding protocol

Mice were anesthetized using Ketalar/Rompun solution intraperitoneally (50 mg Ketalar and 16 mg Rompun per kg body weight) and placed on a heating plate prior to surgery. One six mm full-thickness punch biopsy wound was set on the back of each mouse by folding the back skin and punching through 2 thicknesses of skin. Wounds were immediately treated with emulsions containing SEC^PBMC^, lyophilized medium or physiological NaCl-solution (15 mice per group). Wounds were left uncovered and emulsions were applied once a day for 3 consecutive days. Wound area was measured in two dimensions with handyman's calipers for the first 3 days until a crust was formed and photographed at the beginning and the end of the experiment. Mice were sacrificed on day 7 and wounds were analyzed with regard to their morphology by Hematoxylin-Eosin (H&E)-staining, and the expression of Ki67 and CD31 was analyzed by specific immuno-stainings.

### Cell culture

Human peripheral blood mononuclear cells (PBMC) from young healthy volunteers were cultured in serum-free UltraCulture Medium (Lonza, Basel, Switzerland). Human dermal Fibroblasts (FB, Lonza) were cultured in DMEM (Gibco BRL, Gaithersburg, USA) medium, supplemented with 10% fetal bovine serum (FBS, PAA, Linz, Austria), 25 mM L-glutamine (Gibco) and 1% penicillin/streptomycin (Gibco). Human primary Keratinocytes (KC) were cultured in KC-growth medium (KGM, Lonza). Endothelial cells (EC) were cultured in EC-growth medium (EGM2 MV, Lonza). All cells were cultured at 37°C, 5% CO_2_ and at 95% relative humidity.

### Generation of lyophilized cell culture supernatant derived from PBMC (SEC^PBMC^)

Fifty ml of venous blood were drawn from 10 healthy volunteers. PBMC were separated by Ficoll-Paque (GE Healthcare Bio-Sciences AB, Stockholm, Sweden) density gradient centrifugation. Cells were washed twice with PBS (Gibco), resuspended in serum-free UltraCulture Medium (Lonza) and cultured for 24 h at a cell density of 2.5×10^6^ cells per ml. After 24 h supernatants were collected by spinning cell-suspensions at 500 g. All supernatants were pooled and dialyzed against ammonium acetate (50 mM, Sigma, Vienna, Austria) for 24 h at 4°C. For dialysis a cut-off of 6–8 kDa of the dialysis-membranes (Spectrum Medical Industries Inc., Los Angeles, CA, USA) was used. The obtained liquid was sterile filtered (Whatman Filter 0.22 µm, Dassel, Germany), frozen at −80°C and lyophilized overnight (Lyophilizator Christ alpha 1–4, Martin Christ Gefriertrocknungsanlagen GmbH, Osterode am Harz, Germany). Lyophilization was performed at −20°C and a pressure of 0.1 mbar. The so obtained powder was reconstituted with the respective media for *in vitro* use or further processed to generate an emulsion for *in vivo* applications.

### Preparation of SEC^PBMC^ emulsions

Lyophilized SEC^PBMC^ was resolved in physiological NaCl-solution (Braun, Melsungen, Germany) to obtain a solution corresponding to an equivalent of the secretome from 2.5×10^7^ cells per ml. As control we used lyophilized UltraCulture medium resolved in physiological NaCl-solution and physiological NaCl-solution alone. One part of the solutions was mixed with one part of a hydrophilic cream base (Ultrasicc®). 100 µl of these emulsions (containing SEC^PBMC^ of 1.25×10^6^ PBMC) were applied to one skin wound per day for the first 3 days. Initially we performed the experiments with the dose used mentioned above and a lower dose (1/5). In this pre-experiment we found similar effects with the higher dose, but the lower dose was not effective. The frequency of application of the emulsion was chosen in agreement with our veterinary.

### Antibodies

All antibodies used are listed in Table 1.

**Table 1:** List of Antibodies.

Antigen	Company	No.	Dilution	
			*Western blot*	*Immuno-staining*
CD31	BD Biosciences^1^	553710	n.d.	1∶50
Ki67	Novus Biologicals^2^	NB500-170	n.d.	1∶50
Ki67	Abcam^3^	AB15580	n.d.	1∶1000
c-Jun	NEB^4^	9165	1∶100	n.d.
phospho-c-Jun	NEB^4^	9261	1∶100	n.d.
CREB	NEB^4^	9197	1∶100	n.d.
phospho-CREB	NEB^4^	9198	1∶100	n.d.
Akt	NEB^4^	2938	1∶100	n.d.
phospho-Akt	NEB^4^	9271	1∶100	n.d.
Erk1/2	NEB^4^	4695	1∶100	n.d.
phospho-Erk1/2	NEB^4^	4376	1∶100	n.d.
Hsp27	NEB^4^	2402	1∶100	n.d.
phospho-Hsp27	NEB^4^	2404	1∶100	n.d.
GAPDH	HyTest^5^	5G4	1∶2000	n.d.
mouse IgG HRP	Amersham^6^	NA931V	1∶10000	n.d.
rabbit IgG HRP	Thermo Fisher^7^	31460	1∶10000	n.d.
rabbit Fluor546	Alexa^8^	A-11035	n.d.	1∶500
rat igG HRP	Amersham^6^	NA9350	n.d.	1∶500

Company addresses:

1 Bedford, MA, USA; ^2^Littleton, CO, USA; ^3^Cambridge, UK; ^4^NEB: New England Biolabs, Beverly, MA, USA; ^5^Turku Finland; ^6^Buckinghamshire, UK; ^7^Rockford, IL, USA; ^8^Eugene, OR, USA. n.d.: not done.

### Histochemistry and immunohistochemistry

For analyses, the complete wounds including 2 mm of the epithelial margins were excised, bisected and immediately processed. Skin wounds were either fixed in 4% formalin overnight and embedded in paraffin, or immediately embedded in optimal cutting temperature compound (TissueTek, Sakura Finetek, Zoeterwoude, The Netherlands), snap frozen in liquid nitrogen and stored at −80°C until further processing. H&E-staining was done on 5 µm thick paraffin sections.

### Staining of paraffin-sections

Ki67 staining was performed on paraffin embedded tissues after antigen retrieval by boiling in a microwave for 5 minutes in citrate-buffer (pH = 6, Dakocytomation, Glostrup, Denmark). Non-specific staining was blocked by incubation with 10% normal goat serum for 1 h. The slides were subsequently incubated overnight in a humidified chamber at 4°C with either a Ki67 antibody or isotype-matched control antibodies (Table 1) diluted in PBS containing 2% bovine serum albumin (BSA) and 10% goat serum. To visualize Ki67, sections were incubated with a rabbit Fluor546 antibody (Alexa) for 60 min. After washing nuclei were stained with Hoechst (Dakocytomation) and the slides were mounted with Fluoprep (bioMerieux, Marcy l′Etoile, France).

### Staining of cryo-sections

CD31 immunostaining was performed on 10 µm thick sections of frozen tissues after fixation in aceton/methanol (1∶1) for 10 min. The staining was performed as described for paraffin-sections without boiling in a microwave. The CD31 antibody and the second step antibody used are listed in Table 1. After washing with PBS, slides were incubated sequentially with an HRP-linked second step antibody in PBS containing 2% BSA and 10% normal goat serum for 1 h, followed by incubation with DAB Chromogen tablets (DAKO, Carpinteria, CA). After washing, nuclear staining was performed by incubation with hematoxylin for 10 sec. Slides were mounted with Fluoprep (bioMérieux).

### In vitro scratch assay

3×10^5^ FB and KC were seeded in 6-well plates. After reaching 100% confluence, cells were washed twice with PBS and scratched using a pipette-tip. The resulting scratch was investigated under the microscope and the area for photographs was marked using a pen on the bottom of the well. Immediately after scratching, the first photograph was taken (initial wound size) exactly at the marked area. Lyophilized SEC^PBMC^ (2.5×10^6^ per ml) as well as lyophilized medium were resolved in the respective medium without additional growth factors and added to the scratch-wounds. After 18 h the same area of the scratch wounds were photographed again and the reduction in wound width was measured using ImageJ 1.45 s software (National Institutes of Health, Bethesda, MD, USA).

### Cell cycle analysis

1×10^5^ FB, KC and EC were seeded in 6-well plates and cultured overnight in their respective growth medium. To synchronize cells they were cultivated in basic medium for 18 hours. Lyophilized SEC^PBMC^ (2.5×10^6^ per ml) and lyophilized control medium were resolved in either DMEM, basic keratinocyte medium (KBM, Lonza) or basic endothelial cell medium (EBM, Lonza) - all without serum and growth factors - and added to the respective cell types for 24 h. Cell cycle analysis was performed according to the manufacturer's instructions (BD Biosciences, Franklin Lakes, NJ, USA). Briefly, after 24 h of treatment, cells were incubated with BrdU (10 µM final concentration, kit-component) for 2 h. After fixation (BD Cytofix/Cytoperm™, kit-component) cells were stained with a fluorescein isothiocyanate (FITC) conjugated anti-BrdU antibody (BD Biosciences, kit-component) for 30 min. After washing cells were stained with 7-AAD (BD Biosciences, kit-component) and immediately analyzed on a FACS-Calibur (BD Biosciences). Gates were set according to the manufacturer's instructions and data were evaluated using the FlowJo software (Tree Star, Ashland, OR, USA).

### Tube formation assay

Twenty-four-well plates were coated with 300 µl of Matrigel (BD Biosciences) and incubated for 1 h at 37°C. 1×10^5^ EC in 1 ml EBM containing 10% FBS with either lyophilized SEC^PBMC^ (derived from 2.5×10^6^ per ml) or lyophilized medium were seeded into duplicate wells and observed 24 h after incubation. EC treated with 100 ng/ml vascular endothelial cell growth factor (VEGF, R&D-systems, Minneapolis, USA) served as positive control.

### Western blot analysis

3×10^6^ FB, KC and EC were seeded in 6-well plates and cultured overnight in their respective growth medium. After removal of the medium cells were washed twice with PBS (Gibco) and cultured in their respective basal medium without growth factors for 3 h. Aliquots of lyophilized SEC^PBMC^ and medium were resolved in the different basal media at a 10-fold concentration. One tenth of this solution was then directly added to the cell cultures. After 1 h cells were washed with PBS and lyzed in SDS-PAGE loading buffer. After sonication and centrifugation, proteins were size fractionated by SDS-PAGE through an 8 to 18% gradient gel (Amersham Pharmacia Biotech, Uppsala, Sweden) and transferred to nitrocellulose membranes (BioRad, Hercules, CA, USA). Immunodetection was performed with anti-c-Jun, anti-phospho-c-Jun, anti-CREB, anti-phospho-CREB, anti-Akt, anti-phospho-AKT, anti-Erk1/2, anti-phospho-Erk1/2, anti-Hsp27, anti-phospho-Hsp27 (Ser15) and anti-GAPDH followed by a HRP-conjugated goat anti-mouse IgG antiserum or a goat anti-rabbit IgG antiserum (all antibodies are listed in Table 1). Reaction products were detected by chemiluminescence with the ChemiGlow reagent (Biozyme Laboratories Limited, South Wales, U.K.) according to the manufacturer's instructions. Bands from 3 independent experiments were quantified using ImageJ software and the mean increase in phosphorylation was calculated in relation to the expression of the respective non-phosphorylated proteins.

### Quantification of blood vessel density

To quantify neo-angiogenesis, the granulation tissue underneath the wounds of each CD31 stained wound from 15 animals in each group was evaluated. CD31 positive blood vessels were counted and expressed in absolute numbers. In addition, wound angiogenesis was analyzed by measuring the percentage of the CD31^+^ area using ImageJ software. The percentage of the CD31^+^ area in relation to the granulation tissue was calculated.

### Quantification of Ki67 positive keratinocytes

High-power images of Ki67-stained sections were used to quantify the number of proliferating KC. Digital images of Ki67-stained slides were taken from the regenerated epidermis at the wound edges of each sample. Ki67-positive KC within the full width of the captured field were counted. Regenerated epidermis was defined by hyperthickening and lack of hair follicles.

### Statistical methods

**Figure 1:**
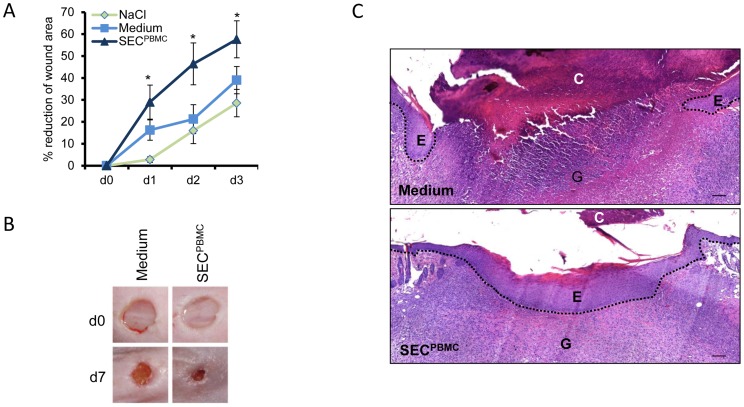
SEC^PBMC^ leads to enhanced wound closure and re-epithelialization. (**A**) Wound areas were measured during the first 3 days after wounding. Treatment with SEC^PBMC^ significantly enhanced wound closure. Error bars represent one standard deviation calculated from 15 animals for each set of values (*: p<0.05). (**B**) Representative photographs from mouse wounds (n = 15 from each group) immediately after wounding and at day 7 after wounding are shown. (**C**) H&E staining of wounds treated with medium or SEC^PBMC^ 7 days after wounding is shown. While medium treated wounds still show a thick crust and little re-epithelialization, SEC^PBMC^ treated wounds are fully re-epithelialized. C  =  crust, E  =  newly formed epidermis, G  =  granulation tissue. Scale bars: 100 µm. One representative animal of 15 is shown.

Statistical analysis was performed using the program Graph-Pad Prism version 5 (GraphPad Software Inc., San Diego, CA, USA). Statistical comparison of two means was performed by using Student's t-test, and comparison of more than two means was performed using analysis of variance (ANOVA) with Bonferroni multiple comparisons post-test. For Figure 1A Kruskal-Wallis testing was used. A p-value <0.05 was defined as the level of significance and is depicted with *.

## Results

### SEC^PBMC^ containing emulsions enhance wound healing in mice in vivo

To study the capacity of SEC^PBMC^ to promote skin wound healing we used the full thickness punch wound model in C57BL/6J mice. We generated emulsions containing either lyophilized SEC^PBMC^, lyophilized UltraCulture medium or physiological NaCl-solution. Six mm punch biopsies were set on the backs of C57BL/6J mice and treated daily with the different emulsions for 3 days. Wound areas were analyzed throughout the healing process until a crust had formed. Compared to an emulsion containing lyophilized medium as well as an emulsion containing NaCl-solution, application of an SEC^PBMC^-containing emulsion strongly enhanced wound closure during the first 3 days (Figure 1A). This advantage in wound closure was still visible at day 7, as shown by representative photographs of the wounds (Figure 1B). H&E staining of the wounds after 7 days revealed that wound healing was more advanced after application of SEC^PBMC^ containing emulsion (Figure 1C). Microscopic examination revealed that the wound gap distance was smaller in the SEC^PBMC^-treated mice and re-epithelialization was markedly enhanced in these animals (Figure 1C).

### SEC^PBMC^ induces migratory capacity of dermal fibroblasts and epidermal keratinocytes

**Figure 2:**
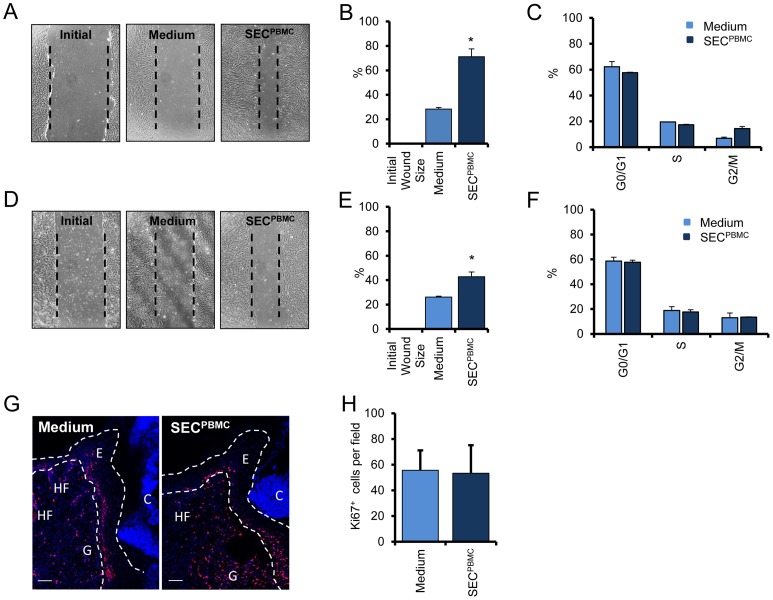
SEC^PBMC^ induces migration of human primary fibroblasts and keratinocytes. Scratch wounds of FB (**A**) and KC (**D**) are shown. One representative experiment of three each done in triplicates is shown. The mean-width of the gaps of nine scratch wounds after 18 h was measured and the percentage of closure for FB (**B**) and KC (**E**) was calculated. (*: p<0.01). Cells cycle analyses revealed no significant differences in FB (**C**) and in KC (**F**). One representative experiment of three each done in triplicates is shown. (**G**) Ki67 staining of medium and SEC^PBMC^ treated wounds showed no significant alterations in proliferating cells. Photographs were taken at the wound-edge of wounds 7 days after wounding. Ki67 staining is shown in red and nuclear staining is shown in blue. C  =  crust, E  =  newly formed epidermis, G  =  granulation tissue HF  =  hair follicle. One representative animal of 15 is shown. Scale bars: 50 µm. (**H**) The mean from 15 animals per group of Ki67 positive cells at the wound edge of one high power image was calculated.

To investigate the capacity of SEC^PBMC^ to induce cell migration on human primary FB and KC the cells were grown to confluency, scratched and treated with lyophilized control medium or SEC^PBMC^ for 18 hours. As shown in figure 2, SEC^PBMC^ significantly induced cell migration in both, dermal FB (Figure 2A,B) and epidermal KC (Figure 2D,E). By contrast, cell cycle analysis revealed no significant changes in cell cycle progression after SEC^PBMC^ treatment in both cell types (Figure 2C,F). This finding was consistent with our *in vivo* observation, where we could not detect a significant increase in proliferating cells in the regenerated epidermis of the skin wounds treated with SEC^PBMC^ containing emulsions, as demonstrated by Ki67 staining (Figure 2G,H).

### SEC^PBMC^ shows strong angiogenic properties in vivo and in vitro

**Figure 3:**
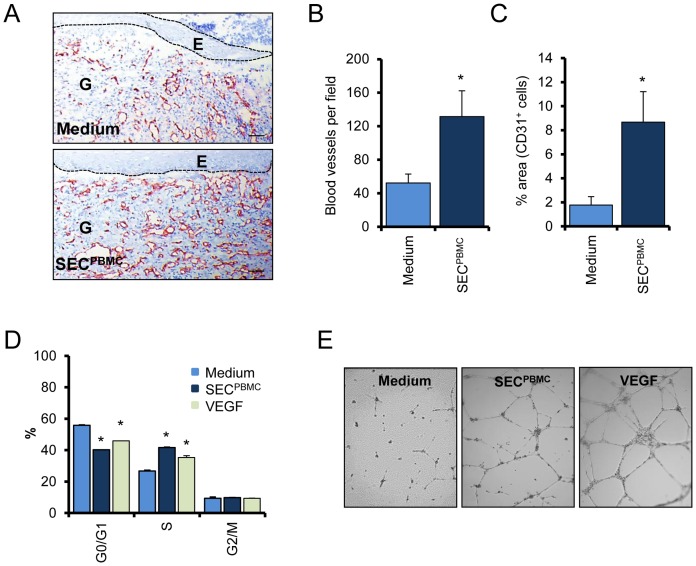
SEC^PBMC^ induces formation of new blood vessels. (**A**) Representative CD31 stainings of wounds underneath the original wound edge are shown. Scale bars: 50 µm (**B**) The numbers of CD31 positive cells underneath the newly formed epidermis were evaluated. The graph represents the mean of 15 animals in each group (*: p<0.01). (**C**) The area of the granulation tissue taken up by CD31^+^ cells was evaluated. The graph represents the mean of 15 animals in each group. (**D**) Cell cycle analysis of SEC^PBMC^ treated microvascular EC shows a strong increase in proliferating cells. VEGF treatment served as positive control. One representative experiment of three each done in triplicates is shown (*: p<0.01). (**E**) A tube formation assay is shown. Compared to medium alone SEC^PBMC^ strongly induced tube formation in a matrigel assay. One representative experiment of three is shown.

Since a crucial event during wound healing is the sprouting of newly formed blood vessels into the wounded tissue, we examined the efficacy of SEC^PBMC^ to induce neo-angiogenesis *in vivo*. Skin wounds, harvested seven days after wounding, were stained for CD31, a specific marker for blood vessels. As shown in figure 3, we found a massive increase in CD31 positive cells in SEC^PBMC^ treated wounds, indicating enhanced neo-angiogenesis (Figure 3A). Blood vessel density in SEC^PBMC^ treated wounds increased from 52±10.7 per high power field in medium treated wounds to 132±31 in SEC^PBMC^ treated wounds (Figure 3B). In addition, morphometric analyses revealed that the percentage of the granulation tissue taken up by blood vessels was markedly increased in the SEC^PBMC^ treated animals compared to the medium treated group (Figure 3C). We further investigated the effect of SEC^PBMC^ on primary microvascular skin EC *in vitro*. In contrast to FB and KC, treatment of EC with SEC^PBMC^ strongly induced proliferation in these cells (Figure 3D). The observed increase in the proliferation rate after SEC^PBMC^-treatment in EC was even higher than that observed after incubation with VEGF (100 ng/ml), which is a known strong promotor of EC-proliferation. In addition to enhanced cell growth, newly formed EC need to reorganize into a three-dimensional tubular structure in a wound healing scenario. We therefore investigated the ability of SEC^PBMC^ to induce tube formation in a matrigel assay system. As shown in Figure 3E, cultivation of EC together with SEC^PBMC^ indeed led to the formation of tubular structures. In comparison, no tube formation was observed in EC cultured with control medium (Figure 3E).

### SEC^PBMC^ treatment leads to activation of signaling cascades involved in cell migration, proliferation and survival

**Figure 4:**
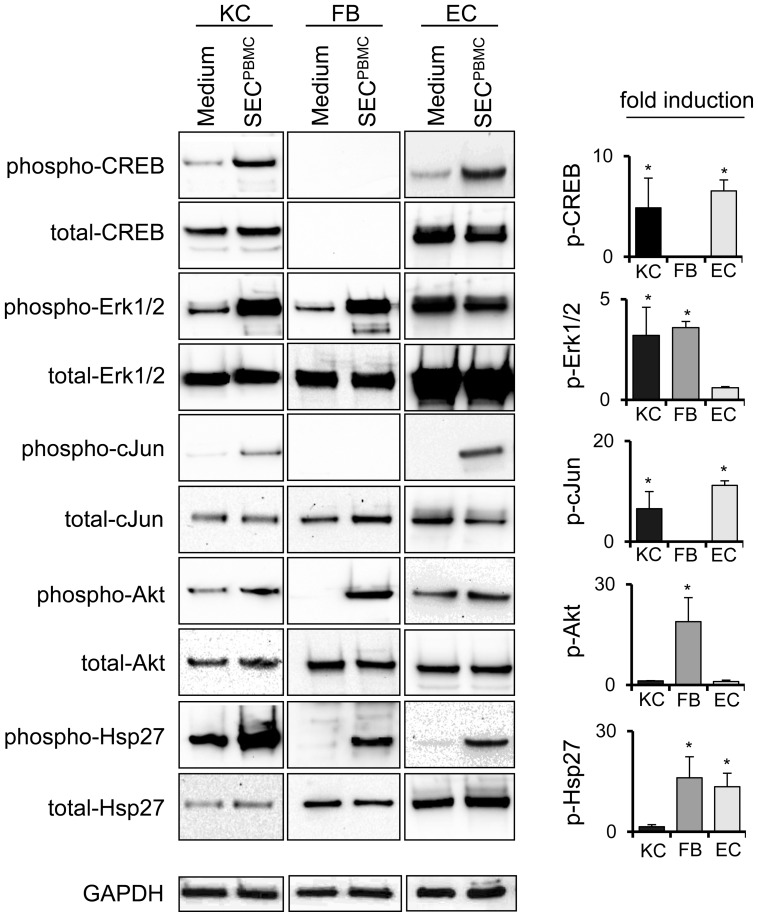
SEC^PBMC^ leads to activation of several signaling cascades. KC, FB and EC were treated for 1 h with medium or SEC^PBMC^. Western blot analyses of several signaling factors are shown. One representative experiment of three is shown. The graphs in the right panel represent the mean band intensity of all three experiments. The increase in expression of the phosphorylated proteins was calculated in relation to the respective non-phosphorylated proteins (*: p<0.01).

To get more information about the underlying mechanisms, we analyzed a variety of signaling cascades involved in cell migration, proliferation and survival. In human primary KC SEC^PBMC^ led to a rapid activation of CREB, Erk1/2, c-Jun, Akt and Hsp27, in human primary dermal FB Erk1/2, c-Jun, Akt and Hsp27 were activated and in dermal microvascular EC SEC^PBMC^ led to the activation of CREB, c-Jun and Hsp27 (Figure 4).

## Discussion

The skin protects the organism against environmental aggressions and microbial pathogens and forms an inside-out barrier preventing fluid loss [1]. Loss of its integrity as a result of injury or illness may cause chronic skin ulcers leading to major disability or even death. Chronic wounds are often difficult to treat, encouraging investigations for new innovative therapeutics that enhance wound healing and tissue regeneration. Recently, cell based therapies have been suggested to be of great advantage in a wound healing scenario. Most of these studies showed positive effects of directly applied highly purified cells to the wounds, which however, are often difficult to isolate and not easily applicable. Increasing evidence is surfacing that all observed therapeutic effects rely on their ability to secrete a cocktail of factors that enhance tissue regeneration, modulate the local environment and stimulate proliferation, migration, differentiation, survival and functional recovery of resident cells [23], [24]. Recent publications have demonstrated that progenitor cells secrete soluble proteins and induce regenerative mechanisms in a paracrine manner [25]–[27]. In addition, also mesenchymal stem cells have been shown to augment the proliferative phase in wound healing that is characterized by angiogenesis, granulation tissue formation, epithelialization and wound contraction [9], [10], [28], [29]. In the present study we demonstrated that lyophilized supernatants of unstimulated cultured PBMC (SEC^PBMC^) are able to enhance wound healing in a mouse model *in vivo*, and induce characteristics of wound healing in human cells *in vitro*.

In a different experimental setting we could previously show that infusion of apoptotic PBMC suspensions in an experimental model of acute myocardial infarction prevented myocardial damage and tissue remodeling [12], , and that this protective effect was also conferred by only applying the secretome of these cells [22]. However, in contrast to stem cells isolated by complicated and time consuming protocols, we used easily obtainable PBMC and showed that one single infusion of PBMC derived “paracrine factors” prevented myocardial damage. We analyzed the secretome of both, apoptotic and living PBMC, and found a variety of highly expressed factors, which have been associated with cytoprotection and tissue regeneration [22]. In line with the work of Di Santo et al., we were not able to block these *in vitro* effects by IL-8, ENA-78, VEGF and MMP9 blocking antibodies [22]. Thus, our data strongly suggest that for tissue protection and regeneration an interaction of several factors is necessary for its full regenerative capacity.

Cell migration and proliferation are rate limiting events in skin wound healing. Here we could show that SEC^PBMC^ not only improved cutaneous wound healing in a murine model, but also induced migration and proliferation of primary human skin cells. Importantly, SEC^PBMC^ treatment of mouse wounds led to a massive sprouting of newly formed blood vessels. Similar angiogenic effects were also found on human dermal microvascular EC *in vitro*, since SEC^PBMC^ strongly induced proliferation and tube formation in these cells. In a further set of experiments we could identify multiple signaling cascades, which were induced after SEC^PBMC^ treatment of different human primary skin cells. Nevertheless, a clear conclusion on the signaling cascades and the initiating factors that might be responsible for the diverse effects in the different cell types cannot be drawn at the present time. Further studies attempting to unravel the factors involved in these processes are ongoing. Currently, we can just speculate on the mechanisms initiated after the treatment with SEC^PBMC^. We have previously determined high amounts of angiogenic factors present in SEC^PBMC^ (VEGF, PDGF, IL-8, ENA-78 and others) [22]. In addition, factors that have been associated with enhanced skin re-epithelialization or that promote wound healing as a chemoattractant to cells of the immune system (eg. MCP-1, RANTES, IL-8) are found in high concentrations [22]. In a recent publication by Lin and coworkers it was demonstrated that toll-like receptor 3 ligand, polyinosinic:polycytidylic acid (Poly (I∶C)), promotes wound healing in a similar mouse model. They further showed that treatment with Poly(I∶C) led to a massive overproduction of chemokines such as MIP-2/CXCL2, MIP-1α/CCL3, MCP-1/CCL2, and RANTES/CCL5, which were responsible for the observed enhanced re-epithelialization [30]. The data by Lin et al. suggest that in our setting these factors might also play an important role for the re-epithelialization of skin wounds. However, in contrast to the study by Lin et al., where a significant effect on wound closure was shown several days after Poly(I∶C) treatment, SEC^PBMC^ induces an immediate wound closure. This might be explained by the fact that SEC^PBMC^ already contains high amounts of cytokines and chemokines, in contrast to other treatments, where production of these factors has to be stimulated first. Together we showed a series of events, all contributing to an improved wound healing. The exact mechanisms however, as well as the responsible factor(s) are still not known. Thus, our data provide the basis for further investigations which will address these points in future studies.

In summary, we have shown for the first time that cell culture supernatants obtained from PBMC (SEC^PBMC^) enhance wound healing in a mouse model and lead to activation of several wound healing associated events in human skin cells. We think that SEC^PBMC^ shows several advantages for the clinical use: 1) The raw material (PBMC) is easily obtainable, 2) It shows minimal or no antigenicity due to cell-free content and 3) it allows “off the shelf” utilization in the clinical setting. Therefore, the formulation and use of SEC^PBMC^ containing emulsions might represent an interesting new option in the treatment of non-healing skin ulcers.
